# Cost-effectiveness of magnetic resonance imaging in coronary heart disease: an economic evaluation using data from the CE-MARC study

**DOI:** 10.1186/1532-429X-15-S1-O7

**Published:** 2013-01-30

**Authors:** John P Greenwood, Simon Walker, Francois Giardin, Claire McKenna, Colin Everett, Jane Nixon, Julia Brown, Mark Sculpher, Sven Plein

**Affiliations:** 1 LIGHT Institute, University of Leeds, Leeds, UK; 2 Centre for Health Economics, University of York, York, UK; 3 Hôpitaux Universitaires de Genève, Geneve, Switzerland; 4 Clinical Trials Research Unit, University of Leeds, Leeds, UK

## Background

CE-MARC was a large prospective study of 752 patients comparing CMR and other tests for the detection of coronary heart disease (CHD). The am of this predefined analysis was to evaluate the cost-effectiveness of diagnostic strategies for CHD derived from the CE-MARC study.

## Methods

Design: Cost-effectiveness analysis using a decision analytic model to compare eight strategies for the diagnosis of CHD. Outcomes are expressed in terms of quality-adjusted life-years (QALYs) and costs are assessed from the perspective of the UK National Health Service (NHS). Population: Patients referred to cardiologists for the further evaluation of symptoms thought to be angina pectoris. Base case characteristics were based upon the CE-MARC study. Interventions: Eight different strategies were considered: 1. CA only. 2. ETT, followed by CA if ETT is positive or inconclusive. 3. ETT, followed by CMR if ETT is positive or inconclusive, followed by CA if the CMR is positive or inconclusive 4. ETT, followed by SPECT if ETT is positive or inconclusive, followed by CA if the SPECT is positive or inconclusive 5. CMR, followed by CA if CMR is positive or inconclusive 6. SPECT, followed by CA if SPECT is positive or inconclusive 7. ETT, followed by CA if positive, or followed by CMR if ETT is inconclusive, followed by CA if CMR is positive or inconclusive 8. ETT, followed by CA if positive, or followed by SPECT if ETT is inconclusive, followed by CA if SPECT is positive or inconclusive. Main outcome measures: Costs expressed as UK sterling in 2010/11 prices and health outcomes in quality-adjusted life-years (QALYs). The time horizon was 50 years.

## Results

Based on the characteristics of patients recruited into the CE-MARC study, only two strategies appear cost-effective for diagnosis of CHD. Both of these included CMR. The choice between a diagnostic strategy in which CMR follows a positive or inconclusive ETT, followed by CA if CMR is positive or inconclusive (Strategy 3 in our model) and a strategy of CMR followed by CA if CMR is positive or inconclusive (Strategy 5 in our model) rests on the threshold cost per QALY gained below which health systems define an intervention as cost-effective. In the NHS, the National Institute for Health and Clinical Excellence (NICE) uses a threshold range (£20,000 per QALY to £30,000 per QALY). The cost-effective strategy is Strategy 3 at the lower end of the range and Strategy 5 at the high end of this threshold range. The results were robust to various scenarios although prior likelihood of CHD requiring revascularisation and the rate at which false negative patients are identified did impact upon the results.

## Conclusions

The economic evaluation results of the CE-MARC study show that using CMR is a cost-effective strategy and support the wider adoption of this modality by health care providers.

## Funding

CE-MARC was funded by the British Heart Foundation (BHF). SP is funded by a BHF fellowship (FS/1062/28409).

**Figure 1:**
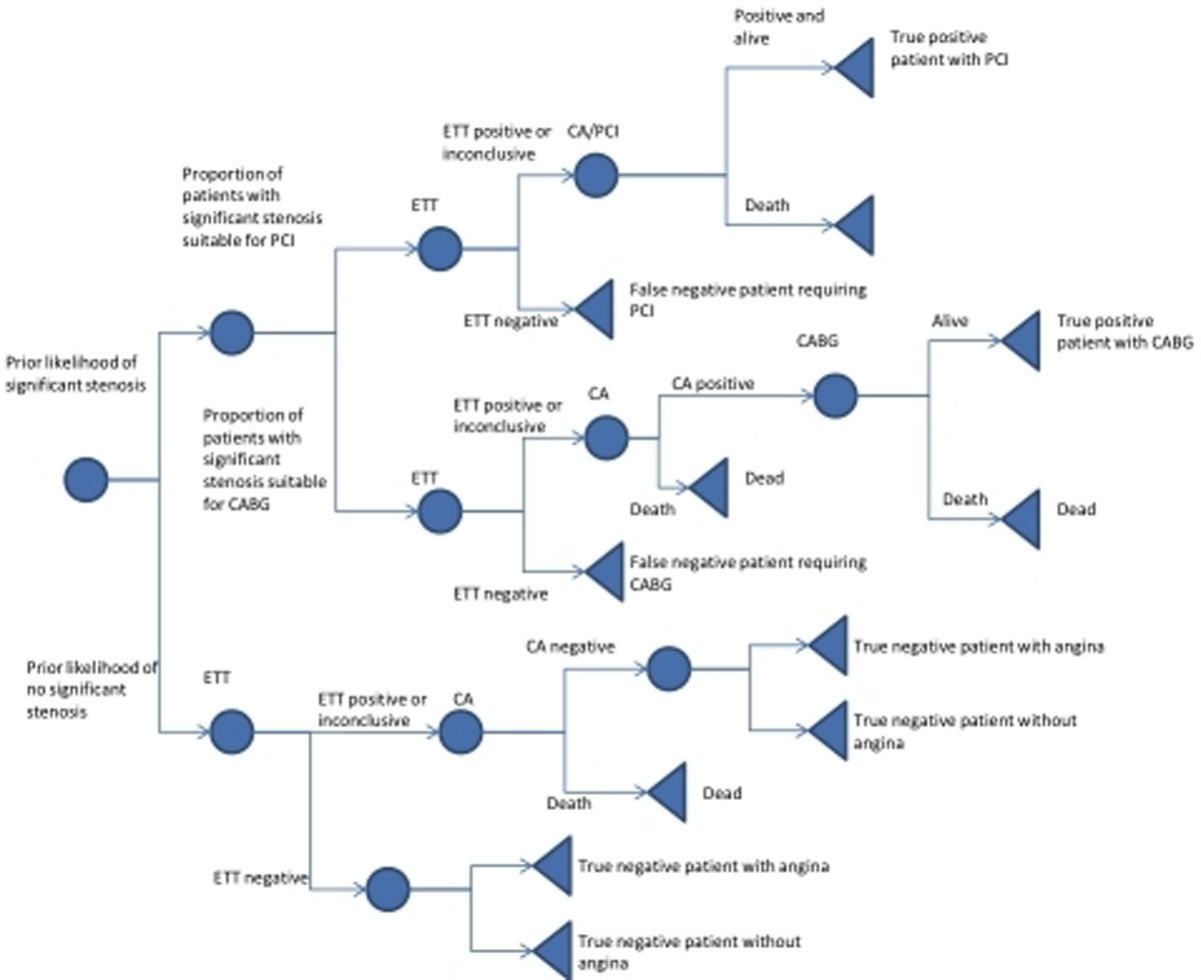
Structure of decision tree using Strategy 2 as an example.

